# Nanomaterials for liver cancer targeting: research progress and future prospects

**DOI:** 10.3389/fimmu.2025.1496498

**Published:** 2025-02-28

**Authors:** Jiahong Xu, Yefu Liu

**Affiliations:** Department of Hepatopancreatobiliary Surgery, Cancer Hospital of Dalian University of Technology, Liaoning Cancer Hospital and Institute, Shenyang, China

**Keywords:** nanomaterials, sorafenib, liver cancer, lenvatinib, gefitinib, targeted therapy

## Abstract

The incidence and mortality rates of liver cancer in China remain elevated. Although early-stage liver cancer is amenable to surgical resection, a significant proportion of patients are diagnosed at advanced stages. Currently, in addition to surgical resection for hepatocellular carcinoma, the primary treatment modalities predominantly include chemotherapy. The widespread use of chemotherapy, which non-selectively targets both malignant and healthy cells, often results in substantial immunosuppression. Simultaneously, the accumulation of chemotherapeutic agents can readily induce drug resistance upon reaching the physiological threshold, thereby diminishing the efficacy of these treatments. Besides chemotherapy, there exist targeted therapy, immunotherapy and other therapeutic approaches. Nevertheless, the development of drug resistance remains an inevitable challenge. To address these challenges, we turn to nanomedicine, an emerging and widely utilized discipline that significantly influences medical imaging, antimicrobial strategies, drug delivery systems, and other related areas. Stable and safe nanomaterials serve as effective carriers for delivering anticancer drugs. They enhance the precision of drug targeting, improve bioavailability, and minimize damage to healthy cells. This review focuses on common nanomaterial carriers used in hepatocellular carcinoma (HCC) treatment over the past five years. The following is a summary of the three drugs: Sorafenib, Gefitinib, and lenvatinib. Each drug employs distinct nanomaterial delivery systems, which result in varying levels of bioavailability, drug release rates, and therapeutic efficacy.

## 1 Introduction

Primary liver cancer ranks among the top five diseases in terms of mortality and the top ten in terms of morbidity worldwide, with its incidence increasing annually (1). This type of cancer primarily includes three pathological types: hepatocellular carcinoma (HCC), intrahepatic cholangiocarcinoma (ICC), and mixed hepatocellular cholangiocarcinoma (CHCC-CCA) (2). Notably, HCC constitutes approximately 90% of primary liver cancer cases (3) and is a leading cause of cancer-related deaths in China. The etiology of hepatocellular carcinoma (HCC) is frequently associated with infections caused by the hepatitis B virus (HBV) and/or the hepatitis C virus (HCV), exposure to aflatoxins, and alcoholic cirrhosis. (4–6). Furthermore, HCC may also arise in individuals with non-alcoholic fatty liver disease (NAFLD), even in the absence of cirrhosis (7).

Early screening for HCC poses several challenges. Currently, abdominal ultrasound and serum alpha-fetoprotein (AFP) tests are more commonly employed than magnetic resonance imaging (MRI) and computed tomography (CT) due to their lower cost, despite being less sensitive to lesions. Additionally, various biomarkers such as AFP-L3, glypican-3 (GPC3), and midkine (MDK) have proven valuable in the diagnosis of HCC (8). Although the necessity of targeted therapy exists, the treatment of liver cell carcinoma remains a continuous challenge. Most of the clinical treatments in the first and second lines combine surgical resection, immunotherapy (9) and targeted therapy. Furthermore, there are additional treatment modalities available, including liver transplantation (10), interventional therapy (2), such as transarterial chemoembolization (TACE) (11), among others. Regarding immunotherapy, vascular generating factors such as vascular endothelial growth factor (VEGF) can inhibit the adhesion of endothelial cells induced by cytokine and the induced endometrial cells that cause endometriosis. Tumors utilize this state to evade immunization. Thus, countering vascular generation is an important part of immunotherapy (12). Commonly used immune checkpoint inhibitors (ICI) include anti-programmed death receptor -1 (PD-1) and anti-programmed death ligand 1 (PD-L1). Immunotherapy frequently combines ICIs with targeted therapies, such as tyrosine kinase inhibitors (TKIs) and VEGFR antagonists, to effectively combat hepatocellular carcinoma (HCC). Additionally, the integration of immunotherapy with phototherapy has demonstrated promising outcomes (13). Not everyone is suitable for immunotherapy, and the presence of resistance to such treatments significantly limits their widespread application. In the context of liver transplantation, challenges including recipient availability and a shortage of donor livers have hindered its large-scale implementation. However, it is anticipated that advancements in 3D printing technology for liver tissue may be achieved in the coming years. Perhaps in the next few years, certain progress will be made in 3D printing of the liver. TACE therapy involves inserting catheters into the blood supply arteries of the tumor and injecting an appropriate amount of emboli at an appropriate speed, resulting in the occlusion of arteries and causing ischemia and necrosis of tumor tissue. Most embolic agents and drugs are combined, simultaneously cutting off the tumor blood supply and enabling the carried drugs to function. The traditional transarterial chemoembolization (cTACE) involves the administration of a mixture of chemotherapeutic agents and liquid lipiodol for embolization. The particle size of sigmal oxidation may cause certain adverse reactions (embolism to normal liver tissue, entering non-target organs, etc.). At the same time, it will also mask the detection of arterial enhancement in tumors and restrict the detection of residual tumors (14). Currently, research has combined intelligent nanomaterials with TACE-related drugs, enabling local tumors to reach higher blood concentrations while significantly reducing drug-related toxicity and hepatic toxicity (14). In summary, the treatment of HCC is still evolving, largely benefiting from advancements in targeted therapies.

Currently, the principle of most targeted drugs used in clinical settings involves inhibiting tumour cell proliferation by targeting specific pathways and angiogenesis. Tumour cell proliferation is intricately linked to metabolism, including nutrient transport and oxygen supply (15), immune recognition factors such as tumour mutation load (16), and the mitotic activity of endothelial cells in capillaries. Tumour cells can promote endothelial cell proliferation, which indirectly affects tumour growth rates. Therefore, tumour cell growth largely depends on the activity of angiogenesis (17). Many of the drugs currently used in clinical applications for liver cancer are multi-targeted. Some of these drugs interfere with the growth cycle of tumor cells. Others act on proteins produced by specific mutations in liver cells.

**Figure 1:**
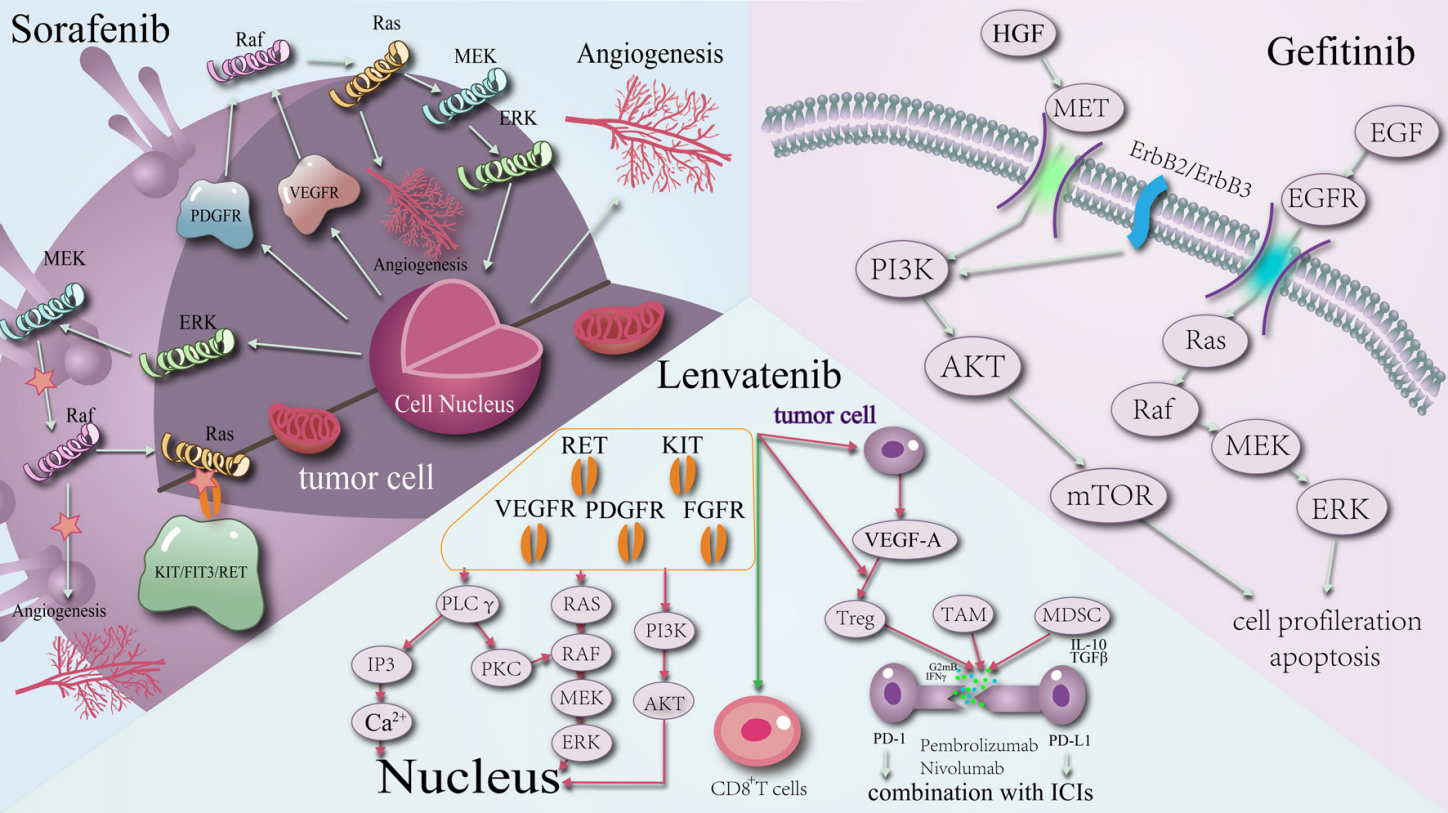
Schematic diagram of the mechanism of action of targeted agents: sorafenib, lenvatinib, gefitinib.

For instance, sorafenib, a commonly used targeted agent, is well-known for its dual mechanisms of action. Firstly, it inhibits tumour cell proliferation by targeting Raf-1, B-Raf, and kinase activity within the Ras/Raf/MEK/ERK signalling pathway. Secondly, sorafenib impedes tumour angiogenesis by targeting platelet-derived growth factor receptor (PDGFR-β) and vascular endothelial growth factor receptor (VEGFR)2, among other proteins. This dual mechanism is not unique to sorafenib; lenvatinib and gefitinib also operate on similar principles (**Figure 1**).

However, targeted drugs are not a panacea. The most common issues are acquired antiangiogenic drug (AAD) resistance and multi-drug resistance (MDR). AAD resistance is variable, as the same tumor may exhibit different microenvironments in distinct locations. MDR refers to the capacity of cancer cells to withstand anticancer agents and can be classified as either primary or acquired. Primary MDR occurs when cancer cells are inherently resistant to a drug. Tumor tissues may contain hundreds or thousands of mutations, and multi-targeting drugs only affect a subset of tumor cells. Consequently, the remaining tumor cells, which lack the target of the drug, proliferate and develop resistance. As the volume of the tumor increases, so does the proportion of drug-resistant cancer cells. Consequently, administering the target drug earlier reduces the likelihood of resistance and enhances therapeutic efficacy. However, most liver cancers are diagnosed at middle to late stages, complicating early intervention. Acquired resistance occurs when cancer cells initially respond to the drug but gradually develop resistance over time and with increased dosage (18).

Multiple factors influence the development of acquired resistance mechanisms. These include the expression of proto-oncogenes and oncogenes, alterations in the tumor microenvironment, drug malabsorption, rapid metabolism leading to lower serum concentrations, changes in T-cell counts, and interactions among cancer cells and between cancer cells and host mesenchymal stromal cells (19, 20). The larger the volume of the tumor, the greater the proportion of drug-resistant cancer cells. Consequently, administering the target drug at an earlier stage reduces the likelihood of resistance and improves therapeutic outcomes. However, most liver cancers are diagnosed at advanced stages, complicating treatment efficacy. Recent advances in multidrug resistance (MDR) research offer promising insights. For instance, N6-methyladenosine (m6A) RNA modification, a reversible process, can regulate the expression of tumor proteins by modulating transporters, metabolic enzymes, and drug targets. This regulation enhances tumor proliferation, growth, and metastasis (21). Additionally, adipose tissue influences drug resistance. Studies have shown that hepatocellular carcinoma (HCC) in fatty livers exhibits more resistance to AAD-sensitive treatments and increased hypoxia during anti-VEGF therapy compared to non-fatty livers (22). To mitigate the emergence of drug resistance and to enhance the efficacy of treatments effectively, many contemporary studies focus on the combination of targeted drugs with various classes of nanomaterials. This approach leverages the targeting capabilities of nanoparticles to deliver therapeutic agents directly to the intended site, thereby minimizing unnecessary loss of efficacy and extending the duration of slow drug release. Additionally, this strategy enhances both the stability and bioavailability of the drug while simultaneously reducing side effects.

## 2 Current status of targeted agents in hepatocellular carcinoma

Historically, sorafenib was the cornerstone of hepatocellular carcinoma (HCC) treatment. However, in recent years, drugs such as lenvatinib and regorafenib have achieved considerable clinical success. More recently, the advent of immune checkpoint inhibitors (ICIs) has revolutionized HCC treatment. The combination of ICIs with other ICIs, anti-angiogenic drugs, targeted therapies, and local-regional treatments has significantly improved the survival rates of HCC patients (3). A common clinical strategy involves the use of anti-angiogenic drugs, targeted agents, and ICIs either alone or in combination to combat HCC. Among the more frequently used targeted agents are multi-targeted tyrosine kinase inhibitors (TKIs) such as sorafenib, lenvatinib, regorafenib, and cabozantinib, as well as the vascular endothelial growth factor receptor 2 (VEGFR2) inhibitor ramucirumab. Commonly used ICIs include atezolizumab, bevacizumab, ipilimumab, nivolumab, and pembrolizumab (**Table 1**).

**Table 1:** Clinical trials of some common targeted agents.

Applied drug principles	Applied drug name	Clinical Data Number (CDN)	Reference
Sorafenib: Inhibitors of various protein kinases (serine-threonine kinase Raf-1, tyrosine kinases, VEGFR and PDGFR) Lenvatinib: Antiangiogenic, oral multikinase inhibitors (targeting VEGFR1-3, FGFR1-4, PDGFR-α, RET, KIT)	Sorafenib/lenvatenib	PhaseIII NCT01761266	(23)
Inhibitors of protein kinases (serine-threonine kinase Raf-1, tyrosine kinases, VEGFR and PDGFR)/PD-L1	Sorafenib/Atezolizumab plus bevacizumab	IMbrave150** ** NCT03434379	(24)
Inhibitors of protein kinases (serine-threonine kinase Raf-1, tyrosine kinases, VEGFR and PDGFR) + PD-1 + CTLA-4 inhibitors	Sorafenib+ Nivolumab+ Ipilimumab	CheckMate040 NCT01658878.	(25)
Inhibitors of protein kinases (serine-threonine kinase Raf-1, tyrosine kinases, VEGFR and PDGFR) + PD-1	Sorafenib+ Nivolumab	PhaseIII NCT02576509	(26)
Inhibition of tumour cell production as well as apoptosis	Sorafenib	PhaseIII NCT00692770	(27)
Tyrosine kinase inhibitors (VEGFR, MET, RET)	Atezolizumab+ cabozantinib/cabozantinib	PhaseIII NCT04338269	(28)
Tyrosine kinase inhibitor (VEGFR, MET, AXL)	Cabozantinib	PhaseIII NCT01908426	(29)
Tyrosine kinase inhibitor (VEGFR, FGFR, RET)	lenvatinib	PhaseIII NCT03905967	(30)
Tyrosine kinase inhibitor (VEGFR, FGFR, RET) +PD-1	lenvatinib+ toripalimab gemcitabine + oxaliplatin (GEMOX)	PhaseII NCT03951597	(31)
Tyrosine kinase inhibitor (VEGFR, FGFR, RET) +PD-1	Lenvatinib+pembrolizumab/Lenvatinib	PhaseIII NCT03713593	(32)
Tyrosine kinase inhibitor (VEGFR, FGFR, RET) +PD-1	lenvatinib+ sintilimab/pembrolizumab/ toripalimab/tislelizumab	PhaseII ChiCTR1900023914	(33)
Regorafenib: multikinase inhibitor (VEGFR, EGFR, FLT3) Sorafenib: Inhibitors of protein kinases (serine-threonine kinase Raf-1, tyrosine kinases, VEGFR and PDGFR)	Regorafenib+ Sorafenib	PhaseIII NCT01774344	(34)
Regorafenib: multikinase inhibitor (VEGFR, EGFR, FLT3) Sorafenib: Inhibitors of protein kinases (serine-threonine kinase Raf-1, tyrosine kinases, VEGFR and PDGFR)	Regorafenib+ Sorafenib	PhaseII NCT01774344	(35)
Regorafenib: multikinase inhibitor (VEGFR, EGFR, FLT3) Lenvatinib: Tyrosine kinase inhibitor (VEGFR, FGFR, RET)	Regorafenib+ Nivolumab (REGONIVO)/Lenvatinib+ Pembrolizumab (LENPEM)	REGONIVO PhaseI NCT 03406871+ RENPEM PhaseII NCT 03609359	(36)
Regorafenib multikinase inhibitor (VEGFR, EGFR, FLT3)	Regorafenib	PhaseIII NCT01774344	(37)
Tyrosine kinase inhibitor (EGFR, PDGFR-β, FGFR,KIT, RET, RAF)	Gefitinib+ cisplatin+ Docetaxel	PhaseIII UMIN000000539	(38)
Tyrosine kinase inhibitor (EGFR, PDGFR-β, FGFR,KIT, RET, RAF)	Gefitinib+ Dacomitinib	PhaseIII NCT01774721	(39)

The clinical selection of drugs for hepatocellular carcinoma (HCC) is generally based on liver function grading and liver staging. The combination of various drugs can lead to a range of adverse effects. For instance, tyrosine kinase inhibitors (TKIs) are frequently associated with hypertension, hand-foot skin reactions, and diarrhea. In contrast, anti-angiogenic monoclonal antibodies often result in hyperproteinuria, hypertension, and hemorrhage. Immune checkpoint inhibitors (ICIs) often lead to immune-related adverse effects, predominantly characterized by elevated transaminase levels (39–41). For patients with advanced HCC, regorafenib may be the preferred option for those with refractory disease. Sorafenib and lenvatinib are recommended when immunotherapy is contraindicated or poses a higher risk of toxic effects. Additionally, regorafenib, cabozantinib, and ramucirumab have shown better efficacy in populations with an alpha-fetoprotein (AFP) level of 400 or higher (42). The use of these targeted agents has proven effective in improving both overall survival and relapse-free survival in patients.

The development of drug resistance remains a significant challenge in the treatment of hepatocellular carcinoma (HCC), necessitating the use of two- or three-drug combinations to enhance therapeutic efficacy and improve patient survival. A major contributing factor to drug resistance in HCC is hypoxia, which influences the effectiveness of drugs like sorafenib by modulating hypoxia-inducible factors (HIFs) and glucose transporters (GLUTs) within metabolic pathways such as glycolysis (43). Despite these strategies, there is currently no effective solution to completely counteract the resistance associated with any single targeted drug. In recent years, nanomaterials have garnered significant attention due to their diverse applications and unique properties. Notably, their excellent physical and chemical characteristics, biocompatibility, and ease of preparation and functionalization make them highly suitable for various biomedical applications. One promising area of research involves using nanoparticles as carriers to deliver drugs, aiming to overcome drug resistance. This approach has shown considerable progress in recent studies. Additionally, surface modification and functionalization of nanomaterials can mitigate potential toxic effects, enhance the stability of nanoparticles *in vivo*, and improve their cell-labeling capabilities (44).

## 3 Several common nanomaterials for HCC

The liver functions as the metabolic hub of the organism, significantly influencing the pharmacokinetics of many orally administered drugs and intravenously delivered nanomedicines through processes such as *de novo* lipogenesis (DNL) (45) and metabolism (46). These processes are intrinsically linked to the livers diverse activities. The livers capacity to catalyze drugs and facilitate oxidation, reduction, hydrolysis, and conjugation reactions enables it to intercept and detoxify harmful chemicals, resulting in the accumulation and eventual metabolism of drugs within the liver (47). However, this can hinder some drugs from reaching their optimal sites of action, thereby reducing bioavailability, therapeutic efficacy, and potentially causing hepatotoxicity.

**Figure 2:**
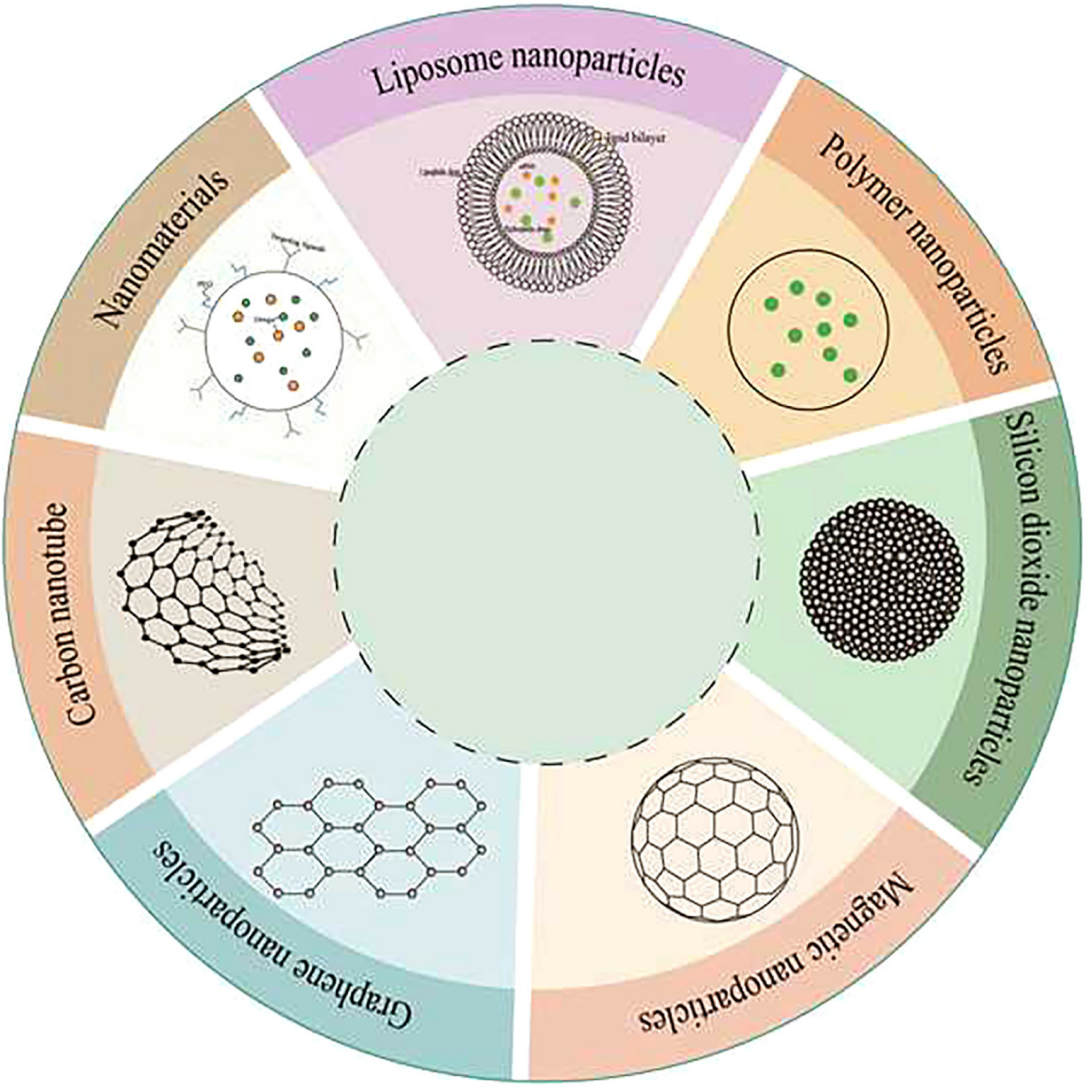
Sketch of several commonly used nanomaterials.

Nanoparticles offer a promising alternative, as they can be more effectively concentrated in tumor areas compared to conventional materials. The liver can uptake nanoparticles through the reticuloendothelial phagocytosis system, which involves Kupffer cells and the deposition of nanoparticles facilitated by the heterogeneity of plasma proteins (2). Different nanomaterials, characterized by their unique structures and compositions (**Figure 2**), play varied roles and have been extensively utilized as carriers in drug delivery systems (DDS). Notably, liposomal nanomaterials (48, 49), silica nanomaterials (50, 51), and graphene nanomaterials (52, 53) are among the most commonly used in applications targeting the liver and hepatocellular carcinoma (HCC).

Despite their potential, nanomaterials are not without limitations. Key challenges in their application include toxicity, target specificity, local drug concentration, unstable drug release rates, and low bioavailability. For instance, some nanoparticles may disintegrate during transport to the target site, resulting in drug loss and diminished efficacy. A summary of common nanomaterials and their characteristics is provided in **Table 2**. Currently, the majority of research on nanomaterials is concentrated on PEG-based nanomaterials and lipid nanoparticle (LNP) formulations. Different types of nanomaterials exhibit a wide range of applications. However, at present, the majority of experiments are concentrated on animal studies, resulting in a deficiency of pertinent data from human trials. Nevertheless, it is undeniable that the potential applications of nanomaterials remain extensive, particularly in the context of targeted therapy for liver cancer.

**Table 2:** Comparison of the characteristics and properties of several common nanomaterials.

Nanomaterials	Advantages	Properties/encapsulated drugs	Reference
Copolymer nanoparticles (PNP)	It accumulates in the liver, enhances drug loading, improves the stability of volatile drugs, and facilitates the slow release of free drugs. It also has FDA approval.	Hydrophobic, suitable for encapsulating lipophilic drugs	(54, 55)
Polyethylene glycol (PEG) nanoparticles	Inhibiting serum protein adsorption on nanoparticle surfaces effectively increased drug accumulation at the target site.	Can be loaded with lipophilic and hydrophilic drugs	(55–57)
Liposome (LNP) nanoparticles	It primarily resides in tissues and organs like the liver, kidneys, and bones, prolonging drug action by inhibiting surface interactions. This enhances the efficiency of oncology drug therapy, offering good stability, biocompatibility, high drug loading, and ease of preparation, while reducing side effects.	Hydrophilic drugs can be encapsulated within and lipophilic drugs can be encapsulated in lipid bilayers.	(55, 57, 58)
Magnetic nanomaterials	It has immunomodulatory effects and can be manipulated *in vitro*, making it highly suitable for MRI.	Its large surface area and high surface-to-body ratio make it suitable for separation in an applied magnetic field and ideal for catalytic systems.	(59, 60)
Carbon nanomaterials (CND)	Excellent optical properties, effective water dispersion, low toxicity, and high biocompatibility.	The small size and short residence time in tumor tissue	(61)
Graphene/graphene oxide (GO) nanoparticles	High thermal conductivity, excellent chemical stability, and minimal scattering of massless charge carriers under ambient conditions.	Possess unique electronic and optical properties, exceptional charge carrier mobility, and remarkable mechanical strength, thermal stability, and chemical stability.	(62, 63)
Metal nanoparticles	Good surface activity, high energy, low toxicity, can be excreted from the body	Unique magnetic, optical, thermal, catalytic and electrical properties	(64, 65)

### 3.1 Liposomal nanoparticles

Liposomes, which are nanoscale hollow structures composed of lipid bilayers, can exist as either monolayers or multilayers. These structures have gained significant attention for their applications in intravenous drug delivery and cancer therapy, with several formulations already approved by the FDA. Liposomal nanoparticles can be categorized based on their diameter and other characteristics into various types, including lipid nanodiscs, small unilamellar vesicles (SUVs), large unilamellar vesicles (LUVs), giant unilamellar vesicles (GUVs), multilayered vesicles (MLVs), multivesicular vesicles (MVs), counter-ionic composite lipid composites, and lipid nanoparticles. One of the key advantages of liposomes is their low toxicity and antigenicity in humans. Additionally, they can encapsulate both hydrophilic and hydrophobic drugs within their lipid bilayers, thereby protecting the drugs from antigenic degradation and enhancing drug utilization (66).

Liposomes can be classified into different types, each with distinct applications and encapsulation capabilities. For instance, large unilamellar vesicles (LUVs) are often utilized in liposomal formulations, while multilamellar vesicles (MLVs) offer enhanced protection for their contents (67). Additionally, various classes of liposomes are designed to encapsulate different types of drugs. Lipid nanoparticles (LNPs), for example, can carry small molecule chemotherapeutic drugs as well as mRNAs. When mRNAs are loaded into LNPs, they are ideally protected during transport to the target organ. Upon reaching the target site, the mRNAs are delivered into the cytoplasm through endocytosis, where they are subsequently translated into functional proteins (68). Zeng et al. Blended cancer cell membranes with thermosensitive liposomes for oxygen carriage and incorporated photosensitizers, near-infrared light fuel, and common fluoride. Through surveillance, in combination with the enhanced permeability and retention (EPR) effect on cancer and the active targeting of cancer cell membranes towards cancer epithelial cells, they exploited the photothermal effect and photodynamic effect to alleviate local hypoxia in HCC while attaining optical imaging (69). The drawback of this prepared complex is that its stability can merely be maintained for approximately 3 days. This article primarily focuses on the efficacy of the nano complex “internally and externally”. There are also articles concentrating on enabling the synthetic nano-complex “self-reliance” to achieve the target effect. For instance, SU et al., encapsulated Sorafeni and ferrite-loose diseases Hemin into a PH-sensitive liposomal liposome. When it reaches the acidic tumor microenvironment (TME), the encapsulated Sorafini, hemoglobin, and hemin are released, increasing the intracellular FE2+ and · OH content, thereby enhancing the apoptotic capacity (70). Researchers prepare composite nanoparticles for the treatment of advanced HCC, but they cannot eliminate metastasis and other similar tumor microenvironments, nor can they determine whether the nano-complexes precisely target the TME of HCC. In terms of drugs, most of the current LNPs are combined with paclitaxel and other targeted drugs. Meanwhile, the efficacy of the drugs is utilized to further generate antibody production (reduce the number of blood vessels and inhibit endothelial cell proliferation) to suppress the growth and metastasis of tumors (71).

The drug loading of liposomal contents primarily relies on the transmembrane PH gradient, which is generated by an internal acidic buffer or a dissociable salt that produces protons. Consequently, the contents are predominantly weakly basic drugs. Research has demonstrated that the incorporation of cholesterol or sphingolipids into liposomes can significantly influence the leakage of their contents. Additionally, the permeability of liposomes is more favorable for hydrophobic drugs while being less permeable to hydrophilic drugs. One notable advantage of liposomes is that the loading of their contents can be conducted independently of the liposome manufacturing process. Furthermore, the drug properties of the contents are dependent on drug concentration. This dependency can be exploited by increasing the concentration of the drug within the liposome beyond its solubility threshold, thereby enhancing precipitation (72).

The delivery of liposomes to cancer cells typically relies on passive targeting, which is based on the enhanced permeability and retention (EPR) effect. This effect necessitates the presence of leaky tumor vasculature. Paclitaxel, a chemotherapeutic agent, is frequently combined with liposomes to enhance its therapeutic efficacy. By leveraging the EPR effect, paclitaxel-loaded liposomes can accumulate more effectively in tumor tissues. This combination has been shown to prolong the survival of animal models by exerting anti-angiogenic effects, such as reducing the number of blood vessels and inhibiting endothelial cell proliferation, thereby inhibiting tumor growth and metastasis (71).

### 3.2 Polymer nanoparticles

Most of PNP are colloidal molecules, which have been approved by two FDAs: polylactide (PLA), poly(lactic acid-glycolic acid) (PLGA), and natural polymers include combinations such as chitosan, gel, and albumin. By altering the ratio of polylactic acid (PLA) to polyglycolic acid (PGA) during the ring-opening polymerization process, PLGA can be obtained in different forms (73). PLGA is utilized as a carrier to prevent the loss of drugs and antigens, and can control the delivery of drugs and antigens to antigen-presenting cells to increase antigen uptake and enhance the immune response (74). The hydrophobic semi-crystalline polyester can be synthesized through ring-opening polymerization or direct polymerization of the lactic acid monomer of biocompatible materials. Its advantages are low toxicity and high biocompatibility (75, 76). PNP can not only enhance the stability of volatile drugs but also improve the sustained-release capacity of free drugs. PNP can act stably as a carrier to carry hydrophobic drugs and continuously release the drug at the target site. Most of the PNP applied in the experiments can incorporate PEG to refine it and increase the circulation time (55, 77).

Studies have designed polymer nanoparticles based on nanometer-loading principle for nanomaterials. PLGA has developed a new lactose-based shell, glycogen (GC) modified nanoparticles (GC@NPs) to enhance the anti-tumor effect of luminin (78). Tong et al. designed a PH-sensitive super-scorcin nanoparticle based on a co-grafting channel for loading sorafini and nuclear factor erythroid 2-related factor 2 (NRF2). The combination of these two drugs induces reactive oxygen species (ROS), iron overload, glutathione (GSH) consumption and promotion of lipid peroxidation, etc., effectively overcoming drug resistance and improving the anti-tumor effect (79). Reversible drugs combined with resistance and targeting drugs are highly promising. Nano-materials can enhance synergy at targeted sites. In recent years, research on polymer nanomaterials has focused more on surface materials, such as LE et al. applying cell-penetrating peptide and Aptamer dual-modified nanomagnetic materials (USila NPs) (UA) and pyromine green (ICG) concentrated nano-drug (USI NPs) on the surface, which can accumulate at the targeted site for fluorescence detection; compared with drugs alone, it can effectively delay tumor cell growth and achieve coordinated treatment of HCC (80).

### 3.3 Silicon dioxide nanoparticles

The advantages of SINPs are evident, and there are numerous applications in medicine. Firstly, although SiO_2_ can cause silicosis, lung fibrosis, and other diseases in peoples lives, SINPs can enter the human body through various means such as inhalation, ingestion, skin contact, and injection. The biocompatibility is high; however, the disadvantage of SINPs is also conspicuous. It depends on the molecular size and dose, and this nanomaterial is highly toxic. Studies have indicated that SINPs can induce lung and liver inflammation, endothelial cell necrosis, the proliferation of alveolar epithelial cells, and are likely to cause pulmonary fibrosis, liver fibrosis, or liver failure. Additionally, other toxic mechanisms of SINPs remain unclear, but it is likely related to NP characteristics (such as size and surface charge). Currently, a silicon-based antioxidant nanoparticle (SIRNP) has been developed. As an ideal oral drug delivery system (DDS), SIRNP not only possesses stable nitrogen oxide free radicals but can also scavenge reactive oxygen species (ROS), and can stabilize under acidic PH and enhance the capacity of loading drugs (81–84).

Based on the characteristics of silicon nanoparticles, Li et al. designed a solution to address the issue of clinical drug resistance and proposed a drug delivery system composed of cisplatin, a silicon dioxide shell, and a PEG-coated surface. Cells combine with nuclear DNA to induce the fundamental principle of apoptosis and utilize the action of “internalization and externalization” to reduce the excretion of cisplatin in the cell, thereby overcoming the role of acquired cisplatin resistance. Additionally, the nanomaterial can also be used as a carrier for loading drugs. When carrying HCC stem cell inhibitors, it reverses internal resistance by inhibiting HCC stem cells (85). The article has an extremely rigorous design of nanoparticles, focusing on reversing drug resistance. Some silicon nanoparticles focus on targeted cancer cells to carry drugs and inhibit HCC growth. The chitosan-coated silicon nanoparticles are loaded into nano-complexes therein. This nano-complex has excellent anti-cancer ability, but compared to other nanoparticles, its cytotoxicity is stronger (86).

### 3.4 Carbon nanotubes

CNT is a molecular graphite carbon tube that is currently widely utilized in drug transportation. Most CNT will be metabolized and eliminated within 24 hours after intravenous injection. CNT can be further refined into multi-wall nanotubes (MWNTs) and single-wall nanotubes (SWNTs). Among them, MWNTs are an attractive type of nano-material. Due to its high stability, good flexibility, large surface area, high biocompatibility, and the ability to improve the capacity of drugs. Compared with other nanomaterials, SWNTs have the advantages of good cell membrane penetration, strong drug-loading ability, long cycle time, etc., which can enhance the efficiency of drug treatment while not accumulating in the liver sinusoidal endothelial cells, thereby helping to reduce toxicity. In addition, CNT co-pyrimidine-based cytidine (CPG) agent has been reported as an effective CPG carrier in neuroplastic tumors. CNT-CPG can combine with glioma, trigger an immune response to inhibit the immune response and inhibit the progression of the tumor (87–89). Carbon nanotubes have been proven to have high apparent diffusion coefficients and can rapidly and deeply penetrate into the tissue, which is difficult for anti-cancer drugs to achieve (90). CHEN et al. designed nanoparticles composed of a gold nanorod core and a silicon dioxide shell, loading sorafeni nibini and the antibiotic gene P53. When the nanoparticles reach the HCC tumor area, the photothermal effect near infrared triggers the release of Sorafenib, which plays a role in coordinating the tumor (91). The article utilizes the rough surface of carbon nanoplastes to carry drugs, resulting in a generally larger particle size of nanomaterials (greater than 200nm). In addition, there are studies that employ the sensitivity of Brucel, using the biosensor to detect liver cell carcinoma (92).

### 3.5 Graphene nanoparticles

Graphene-based Materials (GBM), encompassing graphene oxide (GO), reduced graphene oxides (RGO), and graphene quantum dots (GQD), have emerged as ideal candidates for bone tissue engineering due to their outstanding biocompatibility, which facilitates cell adhesion and proliferation. The remarkable properties of GQD, such as high water solubility, excellent light/PH stability, and elevated reactive oxygen species (ROS) generation, are highly favorable for its application in photodynamic therapy (PDT). The delivery approaches of Graphene-Family Nanomaterials (GFN) encompass oral administration, intravenous injection, and intra-abdominal injection. GFN can accumulate and induce impairments in tissues like the lung, liver, and spleen by traversing multiple barriers, including the blood-brain barrier. Prolonged utilization of GNF can give rise to adverse reactions, such as inflammatory responses, cell necrosis, and DNA damage. Analogously, the adverse effects of GFN are also associated with particle concentration, size, and configuration (93–95). Based on the fundamental characteristics of graphene nanomaterials, Wu et al. devised a dual-targeted oxidized and reduced graphene membrane capable of detecting HCC and its circulating tumor cells (CTCs) that can detect HCC and its spread to the bloodstream. The asialoglycoprotein receptor (ASGPR) can also target the epithelial cell adhesion molecule (EPCAM), and the quenching agent and polymer nanoparticles are modified on its surface. Compared with traditional testing methods, the composite is more targeted and accurate, and is more suitable for the early diagnosis and treatment of HCC (96).

### 3.6 Magnetic nanoparticles

Magnetic nanoparticles (MNPs) are composed of mixtures or pure metals of metals and polymers. They are commonly utilized in PTT, PDT, MRI, magnetic hyperthermia therapy (MHT), or magnetic particle imaging (MPI). It is worthy of mention that compared with MRI, MNPs can be targeted to the tissue site to enhance the image contrast of the targeted tissue. In addition, the nanoparticles that constitute MNP can be further functionalized, and there will be numerous diverse applications. Another advantage of MNPs is that the particles are ferromagnetic/ultra-paramagnetic, which can perform magnetic manipulation in the external magnetic field, thereby enabling them to act on the target site and improve patient compliance. By using magnetic nanoparticles in the drug delivery system, the therapeutic agent can be attached to magnetic particles or nanoparticles, or it can be encapsulated in magnetic particles or nanoparticles. Through the treatment of the magnetic core and polymer or metal coating in the particles, it acquires specific functions. Similar to other nanomaterials, the chemical composition, size, shape, and magnetic behavior of MNPs are also important criteria for determining its biomedical application (55, 97, 98). In addition to removal of magnetic nanoparticles, some studies are also used for magnetic therapy. For example, NASRIN and others covered the radiation gold (198au) on the surface of the nanometer particles of the supercultivated iron (magnetite) nanometer to obtain the core shell nanoparticles (SPION@AU). HCC tumor cells were killed (99). But this method is large and inconvenient to control. GUO and others developed a magnetic metal organic framework (MOF) with homologous tumor cell coating, which can make the tumor blood vessels normally and reduce immunosupply. IV (AS) to regulate the abundance and activity of tumor infiltrating the lymphocytes (TIL), and the coordinating PD-1 inhibitor will provide an immunotherapy for HCC tumors (100).

### 3.7 Gold nanoparticles

Compared with other nanoparticles, metal nanoparticles possess unique physicochemical characteristics such as magnetic, optical, thermal, catalytic, and electrical properties. Metal nanoparticles can not only activate the immune response or improve targeted immune drugs to enhance the autoimmune confrontation against tumors but also directly intoxicate the tumor cells by mediating the reorganization of the extracellular matrix. In addition, metal nanoparticles can also enhance the anti-tumor effect by altering the tumor microenvironment (TME), such as interacting with Toll-like receptors to initiate the inflammatory polarization of macrophages. Subsequently, T cells can be activated by macrophages or metal nanoparticles to achieve anti-tumor cytotoxicity. Metal nanoparticles are often combined with chemodynamic therapy (CDT), photodynamic therapy (PDT), photothermal therapy (PTT), and other applications to enhance the anti-tumor effect.

As a precious metal, gold nanoparticles possess controllable shapes and characteristics. Currently, GNP is fabricated as a biosensor. The application of GNP in cancer treatment largely depends on its ability to penetrate tumor tissue. A more significant application is the combined use of GNP and PTT. In tumor cells within the body, GNP can be converted into thermal energy after near-infrared (NIR) laser exposure (700-1000 nanometers) in the biocontrol process, killing cancer cells and reducing the number of tumors. Studies have confirmed that smaller GNP (below 6nm) can be filtered through glomerular filtration, thereby being excreted from the body, and ultra-small gold nanoparticles can be completely cleared through the liver and kidneys (65, 101, 102).

The clinical application of gold nanoparticles is not only in anti-tumor therapy but also as a contrast agent. Sood et al. have designed a nanometer oxide-gold core-shell structure, which can actively target mitochondria through α-ketoglutarate. Under gamma rays, reactive oxygen species (ROS) and cell debris can be increased, significantly reducing the cell viability of liver cancer cells; it can also be used as a contrast agent for magnetic resonance imaging (MRI)/computed tomography (CT) (103). Wang et al. believe that the radiation absorption efficiency of gold nanoparticles is reduced and the surface area is limited, which has limited their application in radiation chemotherapy. They used the gold-mesoporous silica nanoparticles of the Janus structure prepared by the sol-gel method to form the Janus structure of silica nanoparticles, enabling its loading of doxorubicin and being PH-sensitive, and modified with folic acid. This nano-complex not only shows the effect of inhibiting the growth of HCC tumors but can also be used for targeted computed tomography (CT) imaging in the diagnosis of HCC (104).

## 4 Nanomaterials in several common clinical drugs

Nanomaterials are increasingly recognized not only as nanocarriers for drug delivery but also for their applications in immunotherapy, chemodynamic therapy (CDT), photothermal therapy (PTT), and photochemical therapy (PDT). These therapies offer significant advantages over the use of nanomaterials solely as carriers. Specifically, CDT, PTT, and PDT are selective in targeting cancer cells, thereby minimizing damage to normal cells. The principles and benefits of these therapies are detailed in the following table (**Table 3**). For the purpose of drug selection, we identified three agents that are frequently utilized in clinical practice: sorafenib, lenvatinib, and gefitinib. According to the guidelines for the diagnosis and treatment of primary liver cancer, sorafenib is recognized as the earliest adopted first-line therapeutic agent for hepatocellular carcinoma, Lenvatinib serves as the first-line therapeutic option for patients with unresectable dvanced hepatocellular carcinoma, primarily applies to individuals with unresectable hepatocellular carcinoma who have not previously undergone systemic therapy (122). Gefitinib, a targeted therapy primarily utilized for non-small cell lung cancer, functions as an epidermal growth factor receptor (EGFR) tyrosine kinase inhibitor. In the context of hepatocellular carcinoma, clinical trials investigating the combination of gefitinib and lenvatinib have demonstrated promising efficacy (123, 124). Consequently, we posit that the application of gefitinib in hepatocellular carcinoma warrants further investigation.

**Table 3:** Several novel therapies are common in clinical practice, many of which are now used in combination with nanomaterials.

Common therapies	Abbreviations	Principles	Advantages	Reference
chemodynamic therapy	CDT	The activation of the Fenton or Fenton-like reaction within the tumor microenvironment facilitates the decomposition of endogenous H2O2, leading to the production of toxic hydroxyl radicals (-OH). These hydroxyl radicals can initiate chain reactions with surrounding organic molecules, causing irreversible damage to DNA, lipids, and proteins. This mechanism is particularly useful for inducing cell death in cancer cells, thereby contributing to effective tumor ablation.	It is highly selective and safe, unaffected by light or oxygen, advantageous in hypoxic tumor deep tissues, and shows potential as a novel green treatment with significant clinical applications.	(105–107)
photothermal therapy	PTT	Photothermal therapy (PTT) is an innovative method for targeting and eliminating cancer cells through the use of photothermal agents (PTAs). These agents, which include carbon nanomaterials and gold nanomaterials, are injected into the body and directed towards the tumor site. Upon reaching the tumor, the PTAs are irradiated with an external light source, typically near-infrared light. This irradiation causes the PTAs to convert light energy into heat energy, effectively destroying the cancer cells in the targeted area.	PTT is advantageous for enhancing the efficiency of local light-based heating and tumor ablation, potentially addressing the sterilization shortcomings of other combined treatments. It is non-invasive, temporally controlled, and has minimal side effects.	(108–111)
photochemotherapy	PDT	The use of a non-cytotoxic photosensitiser (PS) or its precursor, when irradiated at a specific wavelength, activates the photosensitising drug at the lesion site. This activation triggers a photochemical reaction that generates singlet oxygen (1O2). The singlet oxygen initiates a chain reaction with surrounding organic molecules, leading to irreversible damage to DNA, lipids, and proteins. This mechanism can effectively target cancer cells, although its efficacy is significantly limited and often ineffective in treating metastatic lesions. Furthermore, the overall efficiency of this approach is also influenced by the systemic anti-cancer immune response of the body.”	Photodynamic therapy (PDT) is a less invasive treatment option that has been approved by the FDA for various cancers, including obstructive oesophageal cancer, obstructive lung cancer, and gastric cancer. Unlike chemotherapy or radiotherapy, PDT is associated with fewer side effects and does not cause significant DNA damage, thereby effectively reducing long-term morbidity. Additionally, PDT does not interfere with future treatment choices for residual or recurrent disease, making it a versatile option in cancer management.	(108, 112–114)
immunotherapy		Cancer immunotherapy has emerged from extensive research into the mechanisms by which tumors evade the immune system. By manipulating these mechanisms, immunotherapy seeks to reactivate the anti-tumor immune response and counteract the pathways that facilitate tumor escape. The primary objective of immunotherapy is to bolster the bodys natural defenses to eradicate malignant cells. Various types of immunotherapies have been developed, including checkpoint inhibitors, lymphocyte-activated cytokines, CAR T-cells and other cellular therapies, agonistic antibodies targeting co-stimulatory receptors, cancer vaccines, oncolytic viruses, and bispecific antibodies.	The FDA has approved it for hairy cell leukemia, metastatic melanoma, and metastatic renal cancer, among other conditions. A more advanced system has been implemented for the first-line clinical treatment of cancer.	(115–118)
sonodynamic therapy	SDT	A therapy combining low-intensity ultrasound (US) with an acoustic sensitizer to target tumor tissue and generate reactive oxygen species (ROS) has emerged as a promising cancer treatment.	An excellent prospective therapeutic strategy offers good tissue penetration and precise temporal and spatial control. SDT therapy, as a minimally invasive tool, can replace antibiotic therapy, reduce systemic toxicity, and decrease bacterial resistance.	(119–121)

### 4.1 Sorafenib

Sorafenib, a multikinase inhibitor approved by the FDA for the treatment of hepatocellular carcinoma (HCC), exerts its therapeutic effects through multiple mechanisms. It inhibits the ERK-MEK-ERK signaling pathway, thereby reducing tumor cell proliferation, inducing apoptosis, and inhibiting angiogenesis by disrupting the growth cycle of tumor cells. However, the extensive application of Sorafenib can lead to significant challenges, including the development of drug resistance and a range of adverse side effects. Common side effects associated with Sorafenib include fatigue, anorexia, diarrhea, skin reactions, and hypertension (125).

Based on the fundamental principles of Sorafenib inhibiting the RAF/MAPK pathway, nanomaterials loaded with Sorafenib can reach the HCC tumor site to enhance their drug efficacy, enhance the targeting of tumor cells, and reduce cytotoxicity. Regarding the tumor microenvironment, reverse resistance, etc. (**Table 4**), currently, there is the first autonomous lipid-based nanocarrier with autonomous therapy. Lipid nanoparticles (LNPs), such as DPPA, can improve the tumor targeting ability with a strong enhanced permeability and retention (EPR) effect, accumulate at high concentrations in the tumor, exert the strong anti-angiogenesis and anti-tumor formation effects of DPPA, reduce toxicity, and increase biological utilization (133).

**Table 4:** Existing studies of nanomaterials used as carriers to deliver sorafenib.

Compound	Nanoparticle	Modes of action	Cellular experiment	Animal experiment	Reference
CNTs-SFN-MCs	Carbon nanotubes (CNTs)	To address poor gastrointestinal absorption and low bioavailability, CNTs-SFN-MCs complexes were developed to enhance drug loading efficiency, thereby increasing the amount of drug that reaches the target site and counteracting drug resistance.	The combination of CNTs-SFN demonstrated enhanced cellular uptake, bioavailability, and anticancer activity compared to sorafenib alone. However, cell flow analyses (MTT) revealed that CNTs-SFN exhibited higher toxicity than sorafenib alone.	Experiments with N-nitrosamine (DENA) -induced Wistar rats demonstrated that treatment with the CNTs-SFN-MCs complex led to greater AFP reduction, preservation of normal liver histology, and prevention of rare nodule formation compared to sorafenib alone.	(126)
Liposome-containing nanoparticle complexes	liposome	Increased drug accumulation at the tumour site.	The *in vitro* cytotoxicity of the complex against HepG2 and Bel-7402 cells was greater than that of sorafenib	Nanoparticle treatment in the H22 tumour-carrying mouse model resulted in a reduction in tumour volume compared to sorafenib-only treatment.	(127)
PFH@LSLP	Oxygen-saturated perfluorohexane (PFH)	Sorafenib treatment activates the CXCR4/SDF-1α axis, exacerbating hypoxia in HCC. This activation promotes tumor progression, invasion, metastasis, and immunosuppression, ultimately increasing resistance to sorafenib.	PFH@LSLP showed sorafenib concentration-dependent toxicity to HepG2 cells in both hypoxic and normoxic conditions.	Fluorescent staining of tumour sections for hypoxia and validated markers in the H22 tumour-carrying mouse model showed that PFH@LSLP enhances anti-tumour effects by reducing hypoxic zones and increasing HIF-1α and CXCR4 expression in PDX HCC tumours.	(128)
(Meo-PEG-S-S-PLGA-G0-C14) (siCFL1/Sor)	PLGA	A key regulator of sorafenib sensitivity, CFL1, was identified, primarily in relation to the remodeling of the cytoskeleton and cell motility. High CFL1 expression enhances serine synthesis and metabolism, as well as the scavenging of sorafenib-induced excess reactive oxygen species (ROS). Consequently, this leads to a diminished sensitivity of hepatocellular carcinoma (HCC) cells to sorafenib.	Enhanced induction of apoptosis and inhibition of MHCC-97L hepatocellular carcinoma cell line formation.	Healthy BALB/c mice and NSG (NOD/SCID/IL2Rγ null) mice were tested, revealing that the complex most effectively inhibited CFL1 expression and suppressed PDX tumor growth. Additionally, *in vivo* serum assays detected only a small amount of toxic residue.	(129)
Ultrasmall lipid nanoparticles (usLNPs) encapsulating SOR + MK-siRNAs	usLNPs	MK-siRNA enhanced the sensitivity of HCC cells to SOR *in vitro*, while SP94 served as an HCC-targeting peptide. usLNP facilitated the delivery of MK-siRNA, SP94, and SOR to the target site, effectively eradicating HCC at a low SOR dose through potent gene silencing and overcoming SOR resistance.	By cellular experiments, it was found that the fabricated usLNPs had no effect on major organs after long-term treatment.	The experiment was conducted using BALB/c nude mice as the model organism. The implantation of the target complex into these mice led to a significant downregulation of alpha-fetoprotein (AFP) and osteoblast-specific protein (OSP) VEGF-1 expression within the tumor tissues. This downregulation was instrumental in reversing resistance to sorafenib (SOR), thereby effectively eradicating established SOR-resistant hepatocellular carcinoma (HCC) in the mice.	(130)
LFC-Sora/Meta-NPs	PLGA-PEG	Methadone, a metabolite of RU486 and a cancer metastasis preventive agent, targets the SDF-1/CXCR4 axis. When combined with sorafenib, it enhances the delivery of both drugs to hepatocellular carcinoma (HCC) cells by specifically recognizing and binding to CXCR4. This combination not only increases cytotoxicity but also modulates the Akt/ERK/p38 MAPK/caspase signaling pathway. Consequently, it inhibits cell proliferation and suppresses potential resistance to sorafenib.	The combination of sorafenib and methadone exhibited significant synergistic cytotoxicity compared to monotherapy, with LFC-Sora/Meta-NPs treatment almost completely inhibiting colony formation in HCC cell lines (HepG2, Huh7, and SMMC-7721 cells).	Female BALB/c nude mice were used in this experiment, in which LFC-Sora/Meta-NPs had the strongest inhibitory effect on tumour growth.	(131)
Curcumin polymer nanoparticle formulations (NFC)	PLGA	Curcumin has demonstrated the ability to reverse multidrug resistance in cancer cells. However, its poor water solubility poses a significant challenge for its therapeutic application. To address this issue, the article explores the use of nanofiber composites (NFC) to enhance the solubility of curcumin. Additionally, the study investigates the combined effect of curcumin and sorafenib on hepatocellular carcinoma (HCC) cells. This combination not only induces apoptosis and cell cycle arrest but also synergistically down-regulates the expression of matrix metalloproteinase-9 (MMP9). The down-regulation of MMP9 is mediated through the NF-κB/p65 signaling pathway, which contributes to the amelioration of drug resistance in HCC cells.	Experiments were performed using Huh7 and MHCCLM3, and MTT results showed that nanocurcumin and/or sorafenib led to increased apoptosis and necrosis, with more necrosis detected after the combination treatment than alone.	Experiments with male thymus-free BALB/c mice revealed that tumours formed mainly in the lungs	(132)

However, research has proved that Sorafenib does not merely rely on inhibiting the RAF/MAPK pathway. For example, Tang et al. analyzed the basic principles of Sorafenib and found that although Sorafenib suppresses the RAF/MAPK signaling pathway, it can activate the PI3K/AKT pathway. This indicates that there is an interaction between the Mapk/ERK pathway and the PI3K/AKT pathway. The potential compensation mechanism presented by the PI3K/AKT pathway can cause Sorafenib resistance in HCC patients. Secondly, the expression of the small nucleolar RNA host gene 3 (SNHG3) is related to the metastasis of HCC and induces the epithelial-mesenchymal transition (EMT) through the miR-128/CD151 level. In addition, knockout of SNHG16 can increase the sensitivity of *in vitro* and HepG2 SR cells *in vivo* (134). Apart from the above signal channel axes, Xu et al. discovered a circular RNA, CircRNA-SORE. They raised HCC cells that had been resistant to Sorafenib, silenced this RNA, and found that Sorafenib-induced apoptosis increased. CircRNA-SORE can act as a microRNA sponge to sequester miR-103A-2-5P and miR-660-3P, competitively activating the Wnt/β-Catenin pathway to induce drug resistance. CircRNA-SORE can be regulated by modulating the N6-methyl adenosine (M6A) level in adenosine. When the M6A level rises, the stability of RNA increases, the level of CircRNA-SORE rises, and the Sorafenib resistance is enhanced (135).

Experiments have demonstrated that nanomaterials can also enhance fluorescent agents while reducing side effects such as Sorafenib resistance. Zhou et al. designed a Prussian blue (PB) nanoparticle (NP). Prussian Blue has been approved by the US Food and Drug Administration. It was combined with a 5.5-long and short metal framework to form SP94-PB-SF-CY5.5 NP. This NP can conduct dynamic monitoring to detect the targeting effect of tumors, exhibit an excellent optical and thermal effect, and alleviate the side effects of Sorafenib. Zhou carried out cell and mouse experiments to confirm that this NP is safe in the body and has a staining effect (136).

### 4.2 Lenvatinib

Lenvatinib is a third-generation anti-tumor angiogenesis targeted drug, specifically a tyrosine kinase (RTK) inhibitor, primarily used in patients with unresectable hepatocellular carcinoma who have not received previous systemic therapy. This drug not only inhibits the kinase activities of vascular endothelial growth factor (VEGF) receptors VEGFR1 (FLT1), VEGFR2 (KDR), and VEGFR3 (FLT4), but also impedes pathological angiogenesis in tumors, thereby aiding in the normalization of blood vessels and inhibiting tumor growth and disease progression (137–139). Notably, lenvatinib can inhibit hepatocellular carcinoma (HCC) cancer stem-like cells through FGFR1-3 signaling pathways, although it does not affect FGFR4 signaling (140). Over the past decade, lenvatinib has supplanted sorafenib as the preferred treatment. Furthermore, combination therapy with immune checkpoint inhibitors (PD-1/PDL-1) is now the first-line treatment for hepatocellular carcinoma, offering improved patient survival and recurrence-free survival compared to sorafenib (141). Lenvatinib is currently widely utilized in clinical settings; however, its low drug utilization and limited efficacy as a monotherapy present challenges. Additionally, the administration of Lenvatinib is associated with a range of adverse reactions, which further complicate its clinical application. These adverse reactions include hypertension, proteinuria, renal failure, and renal insufficiency. Cardiac dysfunctions such as congestive heart failure, cardiogenic shock, and cardiopulmonary failure have also been reported. Hepatotoxicity is another concern, manifesting as increased blood bilirubin, elevated levels of aspartate aminotransferase and alanine aminotransferase, hypoalbuminemia, hepatic encephalopathy, increased y-glutamyltransferase, and elevated blood alkaline phosphatase. Furthermore, patients may experience arterial thromboembolism, diarrhoea, and hypocalcaemia. These complications underscore the need for improved therapeutic strategies to enhance the efficacy and safety profile of Lenvatinib in clinical practice (137, 142, 143).

Lenvatinib has been extensively studied for its role in modulating signal transduction pathways, thereby directly or indirectly influencing lenvatinib resistance. Wang et al. discovered that elevated FZD10 expression promotes hepatic hematopoietic stem cell expansion and lenvatinib resistance. This process is mediated by METTL3-dependent N6-methyladenosine methylation of FZD10 messenger RNA, which enhances hepatic hematopoietic stem cell self-renewal and metastasis through the activation of β-catenin and YAP1. The activation of the FZD10-β-catenin/YAP1 axis in hepatic stem cells is associated with poor prognosis, as it promotes self-renewal, tumorigenicity, and metastasis. Additionally, the FZD10-β-catenin/c-Jun axis transcriptionally activates METTL3 expression, establishing a positive feedback loop. Crucially, the FZD10/β-catenin/c-Jun/MEK/ERK axis determines the response of hepatocellular carcinoma (HCC) cells to lenvatinib treatment. Notably, treatment of lenvatinib-resistant HCC with adenoviral or β-catenin inhibitors targeting FZD10 has been shown to restore the lenvatinib response (141). Numerous researchers have explored the conjugation of lenvatinib with metal nanomaterials to enhance its drug utilization, minimize toxic side effects, and increase its overall efficacy. Among these, gold nanomaterials have shown significant promise. When conjugated with lenvatinib, gold nanomaterials can serve dual functions: they facilitate bioimaging and enable photothermal therapy (PPT). This dual functionality is particularly advantageous in cancer treatment, where it can be applied in various therapeutic modalities such as immunotherapy, chemodynamic therapy, photothermal therapy, and photochemotherapy.

The loading of nanomaterials is intended to enhance the accumulation of drugs in targets and augment the utilization of drugs; the drugs co-loaded simultaneously can also reverse drug resistance. For instance, Qi et al. proposed a novel drug delivery nanoparticle (CAL@PG) designed to enhance drug accumulation at the target site and improve drug utilization, thereby counteracting potential drug resistance. This nanoparticle encapsulates ultrasmall copper sulfide nanocrystals (Cu2-xS NCs) and ultrasmall gold nanoparticles (AuNPs) within galactosamine-conjugated poly(lactic-co-glycolide) (PLGA). The unique properties of CAL@PG allow it to exploit the tumor microenvironment (TME) and facilitate the rapid release of lenvatinib at elevated temperatures induced by the near-infrared II (NIR-II) photothermal effect of Cu2-xS NCs. Furthermore, the elevated temperature, regenerated hydrogen peroxide (H2O2), and lower PH characteristic of the TME drive the reaction towards the production of toxic hydroxyl radicals (-OH). This combination therapy not only significantly enhances the efficacy of lenvatinib but also provides a versatile delivery system for the drug, thereby enriching the nanoparticle-enhanced multimodal synergistic treatment paradigm for hepatocellular carcinoma (HCC) (144).

Lenvatinib, in addition to its application in hepatocellular carcinoma (HCC), is also utilized for targeting cholangiocarcinoma (CCA). Zhou et al. developed a novel delivery system by combining polyethylene glycol (PEG) and the target molecule folic acid (FA) with mesoporous silica nanoparticles (mSiO2) to load Lenvatinib and bufalin (Le/Bu@mSiO2-FA). This innovative approach aims to enhance the targeting of cholangiocarcinomas and offers a potential strategy for reversing multidrug resistance. To validate their hypothesis, Zhou et al. conducted experiments using a human CCA cell line (9810 cells) and a CCA tumour-carrying mouse model. The results demonstrated that Le/Bu@mSiO2-FA significantly impaired CCA cell viability, migration, and invasion *in vitro*, and inhibited tumour growth *in vivo*. Furthermore, biosafety assessments revealed no obvious pathological changes in the heart, liver, kidney, and other organs of the treated mice, indicating minimal toxic side effects. These findings suggest that Le/Bu@mSiO2-FA could be a promising therapeutic approach for CCA with a favorable safety profile (145).

### 4.3 Gefitinib

Geffitinib is a selective epidermal growth factor receptor (EGFR) tyrosine kinase inhibitor, and its target is EGFR. It can not only compete for the EGFR-TK catalytic region MG-ATP binding site to block its signal transmission, but also inhibit the activation of filamented primary activated protein kinase, promote apoptosis, and simultaneously inhibit the formation of tumor blood vessels (146). Gefitinib is a third-line therapy drug for non-small cell lung cancer (NSCLC) approved by the FDA. At the same time, Gefitinib can also improve lung fibrosis (147). For patients with non-small cell lung cancer who have failed chemotherapy with platinum-containing regimens and DOCETAXEL, Gefitinib is often used for the treatment of advanced liver cancer. Although gefitinib is primarily utilized in the treatment of non-small cell lung cancer (NSCLC) and cervical cancer, we posit that lenvatinib also holds significant potential for application in liver cancer. The mechanisms of action of targeted therapies exhibit considerable similarity between NSCLC and hepatocellular carcinoma (HCC). Furthermore, the nanomaterials employed for encapsulating these targeted agents are largely analogous, predominantly consisting of liposomal nanoparticles. Consequently, we contend that experiments involving nanomaterial-mediated delivery of gefitinib in NSCLC may also be applicable to HCC. Geffitinib is usually white powder, which is slightly soluble under PH 1; its solubility drops rapidly in the upper part of the stomach, especially at PH 4-6, and is almost insoluble above PH 7. The degree of dissolution in gastric juice weakens the onset, biological utilization and therapeutic activity. The complications of Gefitinib are mainly adverse reactions such as rash and liver damage (148). Currently, Geffitinib faces significant clinical challenges in its application. If patients use it for a long time, drug resistance will form, hindering the efficacy of the drug. To overcome these difficulties and reduce the drug toxicity of cancer-targeted drugs, researchers have made good progress by combining Gefitinib with nanomaterials instead of using Gefitinib alone.

To combat the resistance of Gefitinib, namely the activation of the EGFR pathway axis, epithelial-interstitial mesenchymal cells, and related influencing factors such as cytotetic cells are mostly involved. Drugs or combined drugs are loaded in nanomaterials. Compared with Gefitinib alone, this drug modality has a better killing effect on tumor cells. At present, most of the research on Gefitinib nanomaterials is focused on non-small cell lung cancer. It cannot be denied that Gefitinib is also frequently used in the treatment of advanced hepatocellular carcinoma. For instance, Liu et al. enhanced cell autophagy-induced cells through the cell autoclave to combat drug resistance. The downstream of the EGFR signaling pathway can be used as a negative regulator in the EGFR signaling pathway. Rapamycin and Gefitinib were combined in shell polytanan nanoparticles (NPs), and anti-EGFR chip doses were applied to design NPs and EGFR NP (NP-APT). They fought together. And experiments were conducted in the H1975 of the NSCLC cell line of Gefitinib-resistant drugs and related mouse lung cancer models. It was found that Gefitinib and Rapamycin were synergistic. The combination therapy significantly weakened the cell activity of H1975 and inhibited tumor growth. Rapamycin has the therapeutic effect of Gefitinib in H1975 cells by inducing autophagy (149). Exosome inhibitor, chloroquine, as an inhibitor that can inhibit the formation of lysosomes, can overcome the self-addiction of anti-drug cells. In recent years, it has also been widely loaded in nanomaterials to combat drug resistance. Zhao et al. believe that Gefitinib can promote the expression of autophagy cell LC3, which is related to its acquisition of drug tolerance. They have prepared chitosan nanoparticles (CS NPs) that can capture Gefitinib and chloroquine to determine whether they have the ability to enhance anti-tumor and overcome drug resistance. And experiments were conducted in the HCC cell QGY cells and QGY/Gefitinib cells (with established drug resistance), and the results show that CSNPs greatly promote the absorption of Gefitinib and enhance the toxicity of QGY/Gefitinib cells. Compared with the control Gefitinib/CQ-NPs, it shows higher suppression rates and apoptosis enhancement. If Gefitinib/CQ-NPs is successful, this model is likely to apply to more drugs that can easily lead to the acquisition of drug resistance, but whether it is applicable to other experiments (150). In the same case, researchers such as Yu combine the anticancer drug Gefitinib and SHRNA to express the chitosan (CS) NP of the plasmid DNA. SHMDR1, as a gene intervention technology, is a genetic intervention technology to enhance the ability of SHRNA to resist DNA enzyme degradation and effectively suppress MDR1 gene expression. The NPS (SHMDR1/GEFITINIB NPS) loaded with SHMDR1 and Gefitinib is prepared to achieve effective co-transportation of genes and antitumor drugs to overcome the multi-drug resistance effect. Gefitinib-resistant Hela cells (with established Gefitinib resistance) were used to conduct MTT and Western blot experiments for verification. The results indicated that NP increased the intracellular accumulation of drugs and restored the sensitivity of cells to the drug, thereby reversing MDR (151). Currently, there is considerable controversy regarding Hela cells, but this should not affect the experimental conclusion.

What interests me more is the research by Cecilia and others. To break the kinase-targeted approach, researchers such as Cecilia and others verify the effective targets of tumors that can be driven by glycoprotein signals. Breaking the targeted target that acts on N-connected sugars can affect all the “curses” of all glycoproteins. Through the directional delivery of nanomaterials, they use small molecular oligosaccharides to partially destroy N-connected glycosylation to transfer Enzyme (OST) inhibitors - NGL-1. Screening 94 cell lines confirmed that there is a significant correlation between OST and epidermal growth factor receptor inhibitors. In non-small cell lung cancer (including HCC827-GR) that is resistant to dystonin inhibitors (TKI), the OST inhibitors maintain the ability to induce cell cycle arrest and proliferation blockade in HCC827-GR. However, after adding NGI-1 in the TKI treatment of epidermal growth factor receptor TKI, the cells of Gefitinib, Erlotinib, or Osimertinib are synthesized. OST inhibition not only destroys the N-connected glycosylation of all epidermal growth factor receptors but also can separate the epidermal growth factor receptor signal from other co-expressed receptors (such as MET) by changing the receptor partitioning. In this way, the tumor growth of HCC827 and H1975, which proves TKI-resistant HCC827 and H1975 heterogeneous transplants through the synthesis and transmission of NGI-1 nanoparticles, is significantly delayed by molecular imaging (152).

### 4.4 Other drugs

In addition to the several anticancer drugs mentioned above and the nanomaterial mechanisms they are applied to, there are many other applications of nanomaterials against drug resistance (**Table 5**).

**Table 5:** The rest of the nanomaterials in liver cancer.

Compound	Nanomaterials	Experimental principle	Reference
FA-SeNPs	Selenium nanoparticles	In the article, folic acid-selenium nanoparticles (FA-SeNPs) were designed as a cancer-targeted nanodrug delivery system for ruthenium polypyridine (RuPOP). These nanoparticles can enter the cytoplasm in a time-dependent manner, facilitating long-term drug release under acidic conditions. The high expression of folate receptors (FAR) in cancer cells significantly enhances the uptake of FA-SeNPs, thereby inhibiting the expression of ABC family proteins and overcoming multidrug resistance in hepatocellular carcinoma (HCC).	(153)
NPSC	Platinum nanoparticles	Bmi-1 is an oncogene that promotes malignancy in various cancers, including hepatocellular carcinoma (HCC). Chemotherapeutic agents, such as cisplatin (CDDP), can treat malignant tumors by targeting Bmi-1. However, CDDP treatment can paradoxically lead to elevated Bmi-1 levels, contributing to the development of HCC resistance. To address this issue, the authors of this paper synthesized a nanocarrier complex, termed NPSC, which comprises siRNA, a platinum nanocore, and calcium phosphate (CaP). This NPSC complex is designed to transport both CDDP and siRNA, thereby enhancing the therapeutic efficacy against HCC. The siRNA component of the NPSC complex increases the sensitivity of cancer cells to CDDP by silencing the Bmi-1 gene, while the platinum nanocore facilitates the delivery of CDDP. The NPSC complex was found to inhibit cancer cell proliferation and improve drug utilization compared to the administration of free CDDP. Additionally, the authors proposed two strategies to combat drug resistance: silencing the Bmi-1 gene and using lipid-calcium-phosphate (LCP) nanocarriers for drug delivery. These findings suggest that the NPSC complex could be a promising approach to overcoming chemoresistance in HCC by targeting Bmi-1.	(154)
NO-DOX@PDA-TPGS-Gal	Unique binding of galactose (Gal) to salivary acid glycoprotein receptor (ASGPR) NP	The nucleoshell structure of the nanoparticle consists of D-α-tocopherol polyethylene glycol 1000 succinate (TPGS) conjugated with galactose (Gal) and polydopamine (PDA). The nanoparticle is loaded with adriamycin (DOX) and a nitric oxide (NO) donor, N,N′-diene-butyl-N,N′-dinitroso-1,4-phenylenediamine (BNN). The authors propose that TPGS and NO function as multidrug resistance (MDR) reversers, inhibiting P-glycoprotein (P-gp)-mediated DOX efflux from HepG2/ADR cells. This results in the formation of a complex, NO-DOX@PDA-TPGS-Gal, which specifically targets hepatocytes through Gal-ASGPR-mediated recognition. Additionally, the complex is designed to respond to a low-PH microenvironment and photothermal conversion of near-infrared light, thereby accelerating DOX release in hepatocellular carcinoma (HCC) cells. This approach is equally effective in HepG2/ADR cells, offering a promising strategy for the treatment of drug-resistant HCC.	(155)
AuNPs-PEG-5FU-FA	Gold nanoparticles (AuNPs)	Many tumors overexpress folic acid (FA) receptors, making FA-coupled gold nanoparticles (AuNPs) an effective targeting mechanism for chemotherapeutic delivery. The presence of FA on AuNPs-PEG-5FU-FA nanoparticles significantly enhances the cellular uptake of these drugs. This increased uptake activates the release of mitochondrial cytochrome C, which subsequently induces apoptosis in cancer cells. Consequently, the incorporation of FA not only targets the nanoparticles to the tumor cells but also enhances the sensitivity of these cells to the chemotherapeutic agents.	(156)
PTXeNBs/siRNA: PTXeNBs/BCL-2 or PTXeNBs/SCR	PTXeNBs, air-core liposomes loaded with PTXs	Combining chemotherapeutic drugs, such as paclitaxel (PTX), with siRNA targeting anti-apoptotic genes like BCL-2, can effectively overcome both efflux pump resistance and apoptosis-related resistance. However, the size of the delivery system poses a significant challenge. To address this, the authors developed multifunctional ultrasound-sensitive bubble-like nanocarriers for the co-delivery of drugs and siRNA. These nanocarriers were created through the heterogeneous assembly of polymeric micelles containing PTX-loaded nanobubbles (NBs) and BCL-2 siRNA complexes. Low-frequency ultrasound forces can enhance the permeability of tumor tissues and disintegrate these complexes (PTXeNBs/BCL-2) into smaller, more diffusible nanoparticles. These smaller nanoparticles serve as potent enhancers for the intra-tumor co-delivery of anti-cancer drugs and siRNA, reaching deeper locations away from the vascular system. Additionally, the co-delivery of the anti-cancer drug adriamycin (doxorubicin, DOX) and BCL-2 siRNA has been shown to improve the chemotherapeutic efficacy against hepatocellular carcinoma (HCC) and overcome drug resistance in HCC.	(157)
Dox/LPL NPs	PEG	Lithocholic acid (LCA) has demonstrated the ability to induce apoptosis in cancer cells through the activation of Casp-3 and Casp-8. However, its clinical application is hindered by its low solubility and short half-life, as well as additional obstacles encountered during *in vivo* use. To address these challenges, the authors propose the use of spherical nanomaterials that mimic red blood cells, which they argue are more advantageous than other shapes. Specifically, they designed Dox/LPL nanoparticles (NPs) that not only counteract angiogenesis to reduce cell proliferation but also decrease the expression of cell cycle-related proteins, such as CDK2 and CDK4, thereby blocking the cell cycle of tumor growth. This dual approach aims to enhance the non-specific targeting of nanoparticle drugs and improve the resistance of anticancer treatments.	(158)

## 5 Discussion

As the incidence of liver cancer continues to rise, the development of new drugs has accelerated, with targeted therapies such as Sorafenib and Lenvatinib undergoing extensive phase I, II, and III clinical trials. In 2021, Donafinil emerged as a more suitable targeted drug for Chinese patients with liver cancer caused by hepatitis, compared to those developed for alcoholic liver cancer prevalent in foreign populations. Concurrently, immunotherapy has gained significant attention and is considered one of the most promising treatments for liver cancer. Its wider application effectively addresses issues such as hypoxia, T-lymphocyte infiltration, fibroblast proliferation, and angiogenesis caused by the tumor microenvironment (TME), while also preventing tumor recurrence and metastasis (159).

The immune receptor inhibitor PD-1/PD-L1 has shown considerable progress in liver cancer treatment. However, despite the gradual improvement in postoperative survival rates for hepatocellular carcinoma, multi-drug resistance remains a significant challenge. Currently, most targeted therapies are administered in combination with other targeted drugs or with immune receptor inhibitors like PD-1/PD-L1 to combat multi-drug resistance. These combination therapies have shown promising results, with preliminary phase III clinical trials indicating improvements in overall survival rates, relapse-free survival rates, and drug efficacy. Nevertheless, effective strategies to manage recurrent drug resistance are still lacking (160).

In addition to these advancements, hybrid nanomaterials have been developed for various applications, including drug delivery, magnetic resonance imaging, enhancement of reactive oxygen species generation in photodynamic therapy (PDT) and chemodynamic therapy (CDT), and the induction of anti-tumor effects and immune responses. Furthermore, 2D materials exhibit great potential in neural repair and regeneration due to their excellent biocompatibility and drug-carrying capacity. For instance, graphene substrates, when used as cellular scaffolds with appropriate electrical stimulation, can significantly enhance cell adhesion and proliferation. Graphene-based nanomaterials (GBNs) can also promote nerve regeneration by delivering drugs to neuronal cells as nanocomposite carriers (161).

Nanomaterials have been extensively developed in the field of medicine due to their unique properties. These materials not only reduce the depletion of drugs during the transport pathway but also increase the accumulation and efficiency of drugs at the tumor site. Consequently, the concentration of drugs in other parts of the body is reduced, thereby minimizing the damage to non-cancerous cells. This selective targeting ensures that other cells in the body remain intact, allowing the immune system to function normally. As a result, the recurrence-free survival rate and overall survival rate are significantly improved. In the context of liver cancer research, the application of nanomaterials extends beyond merely transporting targeted drugs to the tumor site. Studies have also explored the delivery of biomolecules such as mRNA and siRNA, as well as other therapeutic agents like photothermal therapy (PTT) and photodynamic therapy (PDT), achieving varying degrees of success.

To address multi-drug resistance, researchers have increasingly focused on the use of nanomaterials as carriers for targeted drug delivery and immunoreceptor inhibitors. These nanocarriers not only enhance the precision of drug targeting but also reduce drug depletion and potentially amplify drug efficacy indirectly. Various nanomaterials have been employed in numerous applications, including lipid nanoparticles (LNPs) and superparamagnetic iron oxide. For instance, LNPs are utilized to transport siRNA for silencing specific liver gene targets (162), while lipid-solid lipid nanoparticles (LSLNs) carry curcumin derivatives (CU1) to enhance pharmacokinetic effects against hepatocellular carcinoma (HCC) (163). Superparamagnetic iron oxide is commonly used as a contrast agent in magnetic resonance imaging (MRI) and has undergone cytotoxicity testing (164, 165). Additionally, nanocarriers transporting adriamycin are used in conjunction with sorafenib follow-up treatments. Nanoparticle albumin-bound paclitaxel (NAB-PTX) has been employed to deliver trastuzumab (T-mab) and pertuzumab (P-mab) for the treatment of HER2-positive primary breast cancer (PerSeUS-BC04) (166). Furthermore, the homogeneous distribution of carrier-free indocyanine green nanoparticles (nanoICG) into iodine lipids shows significant promise for the precise identification of lesions and the integration of diagnosis and treatment, indicating substantial potential for clinical applications (167).

The application of nanomaterials as carriers for drug delivery has significantly enhanced drug utilization and biocompatibility, reduced drug depletion, and enabled precise targeting, thereby effectively mitigating the occurrence of multidrug resistance. The integration of nanomaterials with therapeutic modalities such as photodynamic therapy (PDT), chemodynamic therapy (CDT), and photothermal therapy (PTT) presents a promising strategy for clinical treatment. This combined approach offers notable advantages, including minimal incision, ease of operation, and reduced side effects, which collectively enhance its clinical utility. For instance, the use of pressurized intraperitoneal aerosol chemotherapy (PIPAC) in conjunction with nanoparticle albumin-bound paclitaxel (NAB-PTX) for the treatment of peritoneal metastases (PM) has demonstrated increased anticancer activity, as evidenced by a phase I clinical trial (168).

Despite the promising potential of nanomaterials, several significant disadvantages remain, particularly as this is an emerging field. Although various materials, such as supramolecular carotenoids, have been developed to enhance the stability of nanomaterials, and preliminary conclusions have been drawn from cell and mice experiments regarding their stability, toxicity, and potential for organ damage, their stability within the human body and their ability to consistently reach target sites remain uncertain. Issues such as the inability to accurately target specific points, the challenges of being phagocytosed by immune cells, or being obstructed by blood vessel and lymphatic walls during transit, and the potential long-term effects on organs like the liver and kidneys, have yet to be effectively addressed. Moreover, there is a scarcity of clinically relevant phase III trials. Therefore, while nanomaterials hold considerable promise and merit further clinical investigation, these unresolved issues necessitate cautious optimism and rigorous research.
